# The Chinese Metabolic Management Centers

**DOI:** 10.1111/1753-0407.13290

**Published:** 2022-06-17

**Authors:** Jianmin Liu, Zachary Bloomgarden

**Affiliations:** ^1^ Department of Endocrine and Metabolic Diseases, Shanghai Institute of Endocrine and Metabolic Diseases Ruijin Hospital, Shanghai Jiao Tong University School of Medicine Shanghai China; ^2^ Shanghai National Clinical Research Center for Metabolic Diseases, Key Laboratory for Endocrine and Metabolic Diseases of the National Health Commission of the PR China, Shanghai Key Laboratory for Endocrine Tumor, State Key Laboratory of Medical Genomics Ruijin Hospital, Shanghai Jiao Tong University School of Medicine Shanghai China; ^3^ Department of Medicine, Division of Endocrinology, Diabetes, and Bone Disease Icahn School of Medicine at Mount Sinai New York New York USA

From 2000 to 2021, global diabetes prevalence increased from 151 million, 4.6% of the adult population, to 537 million, 10.5% of the adult population, with predicted increase to 643 million in 2030, 11.3% of the population, and to 783 million, 12.2% of the adult population, by 2045.^1^ How will this burgeoning group be treated?

Current studies suggest that medical care is not usually optimized for people with diabetes. Of 733 older persons in Vietnam with type 2 diabetes and high cardiovascular disease risk, 11% achieved low‐density lipoprotein (LDL) cholesterol goal, and 51% achieved glycosylated hemoglobin (HbA1c) goal.^2^ In a similar study of 465 adults with diabetes in Brazil, 46% achieved HbA1c goal, 51% blood pressure goal, and 40% LDL cholesterol goal.^3^ In an Iranian study of 2008 persons with diabetes, 78% had HbA1c <8%, and 87% had blood pressure <140/90.^4^ In a Taiwan survey in 2011 of 5599 adults with type 2 diabetes, 35% had HbA1c <7%, 38% had blood pressure <130/80, and 56% had LDL cholesterol <100.^5^ In population samples of persons with diabetes in India and Pakistan in 2016, 30% had HbA1c <7%, and 45% had LDL <100.^6^ From 1999 to 2007 to 2016, US NHANES (National Health and Nutrition Examination Survey) population surveys showed an increase in prevalence of HbA1c ≥8% from 28% to 26% to 20%, in prevalence of blood pressure <130/80 from 42% to 49% to 51%, and prevalence of LDL <100 among persons with diabetes increased from 32% in 1999 to 44% in 2005 to 51% in 2008 and to 53% in 2012.^7^

In a study of 2844 persons with diabetes at high cardiovascular risk in Italy, 56% had LDL cholesterol above minimum LDL goal of 100 mg/dL, and 90% had levels above the more aggressive goal of 55 mg/dL, but, for 35% of these, their physician incorrectly identified cardiovascular risk, suggesting LDL cholesterol targets that were higher than those recommended by guidelines.^8^ This represents what has been termed “therapeutic inertia,” the lack of treatment advancement despite treatment goals not having been attained. Using The Health Improvement Network (THIN) database of 254 925 people in the United Kingdom with incident type 2 diabetes followed for 5.3 years, two‐thirds had dyslipidemia and two‐thirds hypertension, but initiation of lipid‐lowering treatment was delayed 11 to 13 months, and antihypertensive treatment was delayed 20 to 22 months after first being noted.^9^ In a dataset of 54 healthcare organizations in the US including approximately 354 000 patients with type 2 diabetes, approximately 28 000 had HbA1c ≥8%, of whom 46%, 27%, 17%, and 12% had no additional glucose‐lowering treatment at 6, 12, 18, and 24 months.^10^At public health clinics treating 522 persons with type 2 diabetes in Malaysia, therapeutic inertia in increasing glucose‐lowering treatment was seen in up to 64% of visits, in increasing antihypertensive treatment in 51%, and in increasing LDL cholesterol‐lowering treatment in 61% of visits.^11^

Approaches to address therapeutic inertia are limited. In a meta‐analysis of 36 studies of interventions by nonphysician providers such as nurses, certified diabetes educators, and pharmacists to address therapeutic inertia in glycemic management, effective HbA1c reduction was only demonstrated at levels ≥9% and demonstrating reductions far from attaining desirable levels.^12^ Rather than considering this to represent an issue of poor quality healthcare, the repeated finding that the majority of persons with diabetes fail to attain the combined HbA1c, blood pressure, and lipid goals generally considered necessary for prevention of micro‐ and macrovascular complications should be reconceptualized as a diabetes management dilemma representing a systems issue with current approaches to delivery of healthcare. To address this, the Chinese National Metabolic Management Center (MMC) was begun in 2016^13^ as a way of creating a structured treatment approach for the management of healthcare for people with diabetes and at risk of developing diabetes in China, where the diabetes prevalence is 12.4% among adults.^14^ Currently, nearly 1500 hospitals, including community hospitals in 31 provinces in China, are involved in the MMC network, with nearly 900 providing care to nearly 900 000 patients. The MMC treatment protocols follow international and Chinese guidelines, addressing targets for HbA1c, blood pressure, and lipids according to patient age, life expectancy, and existing chronic complications.

Recently, data from 10 MMC centers were analyzed and published in the *Journal of Diabetes*.^15^, ^16^, ^17^, ^18^ MMC real‐world data have been used to explore effective and feasible hierarchical strategies for diabetes management, such as those for initiation of insulin treatment for patients failing to achieve glycemic control with non‐insulin medications.^17^ Analysis of outcome among 19 908 patients with type 2 diabetes with mean follow‐up duration 20.1 months showed that greater visit frequency significantly improved metabolic outcomes, particularly among those with younger age and worse baseline glycemic hemoglobin.^18^ Other studies from the MMCs showed an association of sedentary behavior with greater levels of carotid plaque formation and association of albuminuria and of low epidermal growth factor receptor (eGFR) with greater degrees of arterial stiffness. This encouraging real‐world evidence emphasizes the importance of lifestyle intervention and metabolic goal achievement in reducing diabetes complications.

Going forward, the MMCs will allow “big data” internet technology application and precision medicine to be used in areas of diabetes management from prediction, diagnosis, and treatment to prognosis.^13^ Artificial intelligence‐based diabetic retinopathy screening may offer a good example.^19^ However, MMC still faces many challenges in the future and has space to improve. We believe that with more intervention studies and evidence from long‐term follow‐up, as well as more clinical translation of new technology approaches in the MMC, further refinement of treatment will allow us to break the cycle of therapeutic inertia that hinders attainment of optimal outcome in the hundreds of millions of persons with diabetes worldwide.

## CONFLICT OF INTEREST

The authors declare no conflict of interest.
